# Thoracic skeletal muscle quantification: low muscle mass is related with worse prognosis in idiopathic pulmonary fibrosis patients

**DOI:** 10.1186/s12931-019-1001-6

**Published:** 2019-02-15

**Authors:** Sung Woo Moon, Ji Soo Choi, Sang Hoon Lee, Kyung Soo Jung, Ji Ye Jung, Young Ae Kang, Moo Suk Park, Young Sam Kim, Joon Chang, Song Yee Kim

**Affiliations:** Division of Pulmonary Medicine Department of Internal Medicine, Yonsei University College of Medicine, 50 Yonsei-ro, Seodaemun-gu, Seoul, 120-752 Republic of Korea

**Keywords:** Idiopathic pulmonary fibrosis, Sarcopenia, Computed tomography, Skeletal muscle, Mortality

## Abstract

**Background:**

Sarcopenia can contribute to negative outcomes in patients with various lung diseases. However, whether sarcopenia affects prognosis in patients with idiopathic pulmonary fibrosis (IPF) has not been reported. Simple measures of muscle mass, derived from chest computed tomography (CT), are increasingly being used to identify patients with sarcopenia. We hypothesized that skeletal muscle mass could be a predictor of prognosis in IPF patients.

**Methods:**

We retrospectively evaluated 180 patients diagnosed with IPF between January 2010 and December 2015 at a tertiary care hospital in South Korea. We measured thoracic muscle volume by using the cross-sectional area (CSA) of the pectoralis, paraspinal, serratus, and latissimus muscles at the 4th vertebral region (T4_CSA_) and the erector spinae muscle (ESM_CSA_) at the 12th vertebral region. CT scans at the time of diagnosis were used for analysis and respective CSA were divided by height squared to normalize for stature. Survival times were estimated with the Kaplan–Meier method and compared with the log-rank test. Multivariate Cox proportional hazards models were performed to investigate relationships between clinical parameters and mortality.

**Results:**

Male patients in the lowest quartile of T4_CSA_ divided by height squared (m^2^) (T4MI) and in the lowest quartile of ESM_CSA_ divided by height squared (m^2^) (T12MI) were more likely to have higher Gender-Age-Physiology Index scores (T4MI, 3.3 ± 1.3 vs 4.0 ± 1.6, *P* = 0.012; T12MI, 3.2 ± 1.3 vs 4.1 ± 1.6, *P* = 0.002). Male patients in the lowest quartile of T4MI exhibited a significantly lower survival rate (*P* = 0.035). After multivariate Cox proportional hazards analysis, T4MI was a significant risk factor for all-cause mortality (HR, 0.955; 95% CI, 0.913–0.998; *P* = 0.041), whereas T12MI was not (HR, 0.980; 95% CI, 0.856–1.121; *P* = 0.766).

**Conclusions:**

Low skeletal mass normalized for stature at the level of 4th vertebrae which can be acquired by quantifying thoracic skeletal muscle on single-slice axial chest CT, may be a strong risk factor for all-cause mortality in patients with IPF.

**Trial registration:**

The research protocol was approved by the Institutional Review Board of Severance Hospital, South Korea (IRB No.4–2018-0454).

**Electronic supplementary material:**

The online version of this article (10.1186/s12931-019-1001-6) contains supplementary material, which is available to authorized users.

## Introduction

Sarcopenia is the progressive loss of muscle mass and strength, which is associated with a risk of adverse outcomes, such as disability, poor quality of life, and death [1]. Sarcopenia can cause negative outcomes in patients with various lung diseases, as well as non-lung diseases, such as cholangiocarcinoma and breast cancer [2–7]. Idiopathic pulmonary fibrosis (IPF) is a specific form of chronic, progressive, fibrosing interstitial pneumonia of unknown cause; it occurs primarily in adults and is limited to the lungs [8]. Indexes of IPF prognosis include symptoms, respiratory function, and imaging, which are important to consider in the clinical progression of IPF [8]. However, the effect of sarcopenia on prognosis in patients with IPF has not been reported.

Several modalities have been used to assess sarcopenia. Bioelectrical impedance analysis, dual energy X-ray absorptiometry, magnetic resonance imaging, and B-mode ultrasound are widely used to quantify both total and local skeletal muscle mass [1]. Measurement of the cross-sectional area (CSA) of skeletal muscles on single-slice axial computed tomography (CT) scans is an alternative method to assess local skeletal muscle mass [9]. Simple measures of muscle mass, derived from CT of the abdominal region, are increasingly used to identify patients with sarcopenia [3, 6, 10, 11]. A limitation of this method for evaluating muscle CSA in chronic respiratory disease is that abdominal CT scans are not typically performed in clinical respiratory assessment. However, correlations have been found between muscle CSA at a single thoracic level on chest CT and total muscle volume in patients with advanced lung disease [12–14].

Rozenberg et al. observed that, at the level of carina, which corresponds to the 4th vertebral region, the CSA of the pectoralis, intercostalis, paraspinals, serratus, and latissimus muscles was associated with frailty markers, such as 6-min walk distance, biceps training volume, quadriceps training volume, and length of hospital stay in lung transplant patients [15]. Zuckerman et al. reported similar observations regarding the CSA at the 4th vertebral region, which was associated with frailty markers [7]. Fuseya et al. observed that, at the lower margin of the 12th vertebral region, CSA of erector spinae muscle (ESM_CSA_), a major antigravity muscle group, was associated with severe airflow limitation, respiratory symptoms, emphysema severity, and mortality in chronic obstructive lung disease patients [12].

In this study, we focused on pectoralis, paraspinals, serratus, and latissimus muscles at the 4th vertebral region and erector spinae muscle at the 12th vertebral region. We hypothesized that skeletal muscle mass, measured by analyzing muscle CSA from chest CT images at the 4th and 12th vertebral regions, could be a predictor of prognosis in IPF patients.

## Materials and methods

### General methods

We retrospectively reviewed medical records of patients who were diagnosed with IPF between January 2010 and December 2015 at one tertiary care hospital in South Korea. IPF was diagnosed according to an official ATS/ERS/JRS/ALAT statement [16]. Diagnosis was confirmed by a multi-disciplinary team consisting of specialists in pulmonary medicine, radiology, and pathology.

**Fig. 1:**
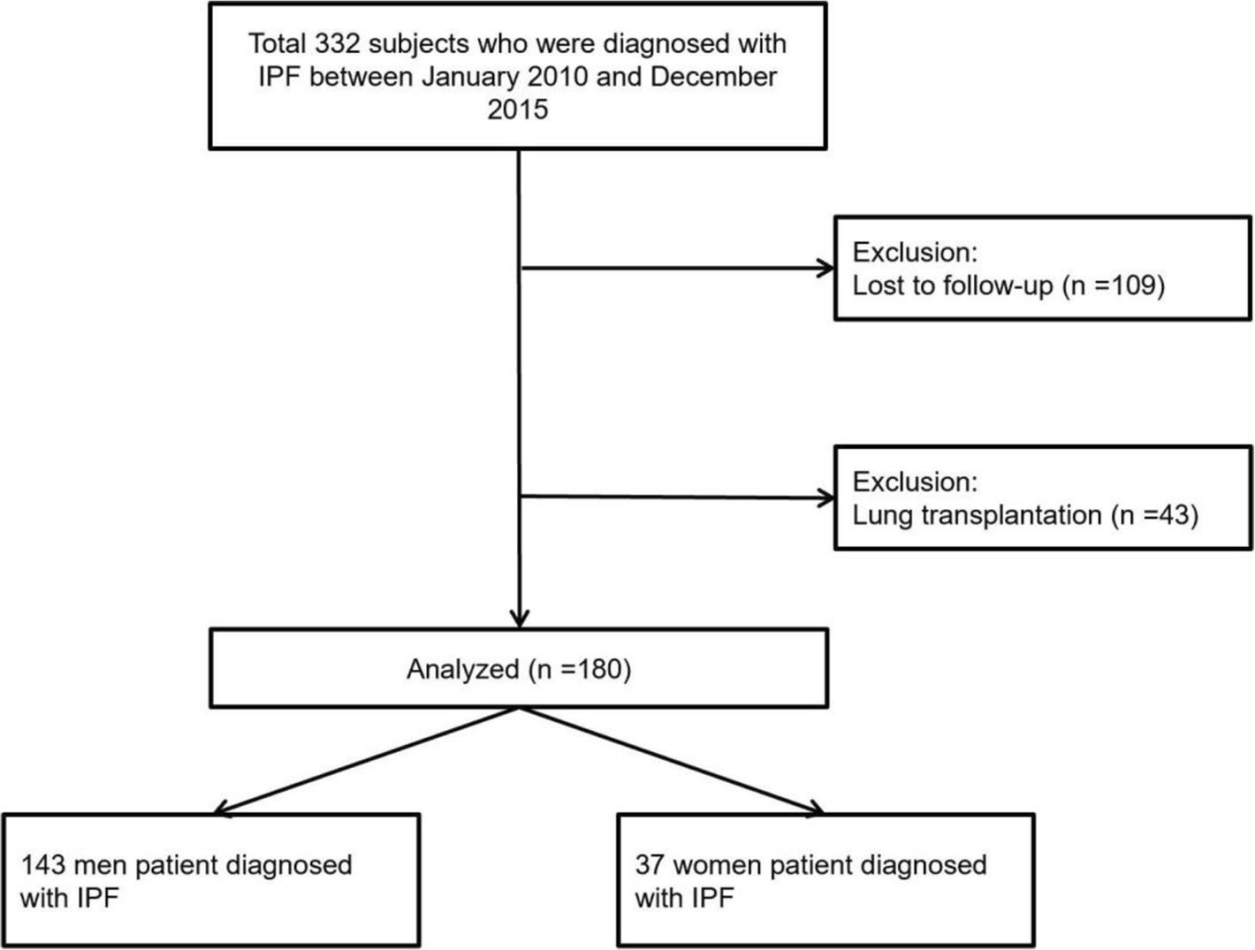
Flow diagram of subjects in this study. Abbreviations; IPF, Idiopathic pulmonary fibrosis; ILD, Interstitial lung disease

The flow diagram depicting the selection process of subjects in this study is shown in Fig. 1. Initially, 332 patients who were diagnosed with IPF were included in the study, selected from our institutional database based on the following criteria: (1) Patients with confirmed IPF by either clinical findings or biopsy; (2) CT scan images at the time of diagnosis were saved in our institutional radiology database; (3) clinical data were available from the medical records. Subsequently, patients who underwent lung transplantation (*n* = 43), and patients who were lost to follow-up (*n* = 109) were excluded. Finally, 180 subjects (143 men and 37 women) were included in the analysis.

Age, smoking history, pulmonary function test results, underlying diseases including diabetes, height, weight at the time of the diagnosis, Gender-Age-Physiology (GAP) Index [17], usage of drugs that may affect skeletal muscle mass [18] (statins, sulfonylureas, glinides) and mortality data were collected for all patients; data regarding thoracic muscle cross-sectional area (CSA) at the level of the 4th thoracic vertebra were available in all patients; data regarding erector spinae muscle CSA at the level of the 12th thoracic vertebra were available in 174 patients. Computed tomography scan images at the time of diagnosis were used for analysis. Follow-up data, including mortality data, were collected until December 2017.

The exact definition of and standardized measurement techniques for sarcopenia have not yet been established at the level of T4 or T12 in IPF patients. Therefore, additional predictor variables were determined based on studies performed by Rozenberg et al. [15] and Fuseya et al. [12] We measured sarcopenia indirectly with thoracic muscle (e.g. pectoralis, intercostalis, paraspinals, serratus, latissimus muscles) CSA, measured at T4 (T4_CSA_). We divided the cross-sectional area of skeletal muscle (cm^2^) at the T4 level by height squared (m^2^) to yield the muscle index at T4 (T4MI, cm^2^/m^2^), normalized for stature. We also sought to measure sarcopenia indirectly with the CSA of the ESM at the T12 level. We divided the CSA of ESM (ESM_CSA_, cm^2^) at the T12 level by height squared (m^2^) to yield the muscle index at T12 (T12MI, cm^2^/m^2^), normalized for stature. The primary outcomes were overall survival, 1-year survival, and 2-year survival after diagnosis. Overall survival was estimated from the date of baseline CT to death or the last follow-up.

### Measurement of skeletal muscle CSA at the level of T4, T12

**Fig. 2:**
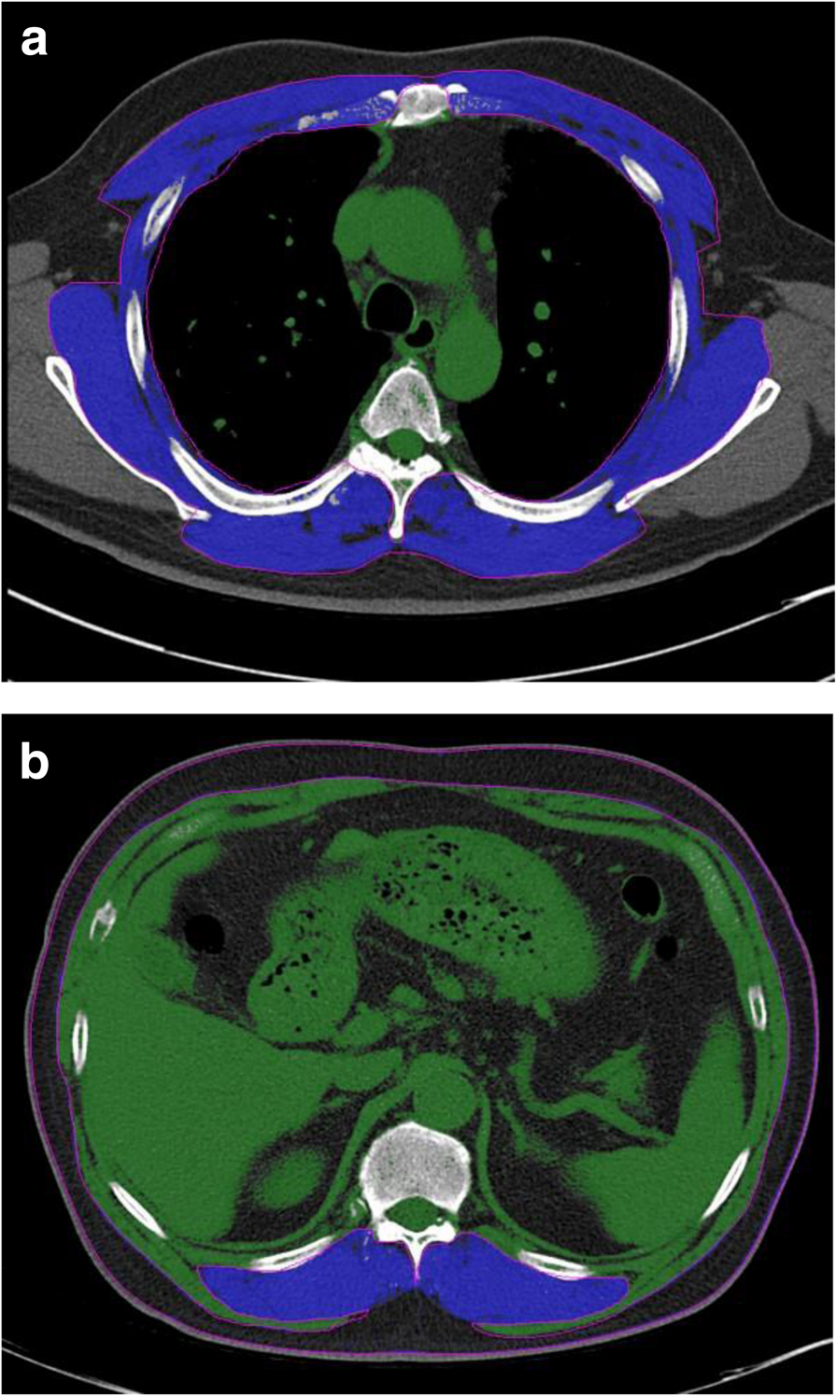
**a**, **b**: Sample computed tomography (CT) scans used to determine muscle area in idiopathic pulmonary fibrosis case subjects. Chest CT examinations were obtained by using 24-detector CT scanners (Somatom Emotion 16; Siemens Healthcare, Erlangen, Germany). Non-contrast CTs were used in the analysis. Quantitative assessment of the CSA was performed semi-automatically with Aquarius iNtuition Viewer (ver. 4.4.11, TeraRecon Inc., San Mateo, CA, USA) (**a**) Axial CT image of the 4th thoracic vertebral region. Pectoralis, intercostalis, paraspinals, serratus, and latissimus muscles are in blue. **b** Axial CT image of the 12th thoracic vertebral region. Erector spinae muscles are in blue

Chest CT examinations were obtained by 16-detector CT scanners (Somatom Emotion 16; Siemens Healthcare, Erlangen, Germany). Non-contrast CTs were used in the analysis. Quantitative assessment of the CSA was performed semi-automatically with Aquarius iNtuition Viewer (ver. 4.4.11, TeraRecon Inc., San Mateo, CA, USA) as shown in Fig. 2. T4 was defined as the slice including the middle of 4th thoracic vertebrae, and T12 was defined as the slice including the middle of 12th thoracic vertebrae; the observer visually identified single cross-sectional images at the levels of T4 and T12, respectively. At the T4 level, we then outlined the borders of the thoracic and back muscles. At the T12 level, we outlined the borders of the ESM. Tissue CSAs in slices were computed automatically by summation of the pixel attenuation of − 30 to + 150 Hounsfield units for skeletal muscle. After applying the threshold method (with a predefined Hounsfield unit threshold) to slices, boundaries between different tissues were corrected manually when necessary. After measuring CSAs, respective CSA was divided by height square to normalize for stature, as used in previous studies. A radiology technician performed measurement of the CSA without access to patient information.

### Statistical methods

Descriptive statistics are reported as numbers with proportions or means with standard deviations (SDs). The continuous variables were tested for normality by using the Kolmogorov-Smirnov test. Chi-squared tests or Fisher’s exact tests were conducted to compare categorical variables between the male and female groups, sarcopenia and normal groups; Student’s t-tests or Mann-Whitney tests were conducted to compare continuous variables between the two groups. Survival times were estimated with the Kaplan–Meier method and compared with the log-rank test. Multivariate Cox proportional hazards models were performed to investigate relationships between clinical parameters and mortality. Variables with a *P*-value of < 0.15 by the log-rank test were included; variables that overlap (e.g., age, gender, forced vital capacity (FVC), and diffusing capacity of carbon monoxide (DL_CO_) in GAP index) were excluded. An adjusted *P*-value < 0.05 was considered statistically significant. All statistical analyses were performed with SPSS version 20.0 (SPSS Inc., Chicago, IL, USA).

## Results

### Baseline characteristics

Baseline characteristics of the study subjects are provided in Table 1. The proportion of male patients was 79.4%. The mean age for all patients was 69.1 years (range, 44–94 years); the mean ages of male and female patients were 68.7 years (range, 44–89 years) and 70.0 years (range, 52–94 years), respectively.

**Table 1:** Patient characteristics

Variable	Total	Men	Women	P-value
Number of patients, n	180	143 (79.4%)	37 (20.6%)	
Age, years	69.1 (44–94)	68.7 (44–89)	70.0 (52–94)	0.214
Height, meters, m	1.6 ± 0.1 (1.4–1.8)	1.7 ± 0.1 (1.5–1.8)	1.5 ± 0.1 (1.4–1.7)	< 0.001
Weight, kg	63.9 ± 10.3 (33.1–97.3)	65.7 ± 10.2 (33.1–97.3)	56.9 ± 7.1 (46.0–73.0)	< 0.001
BMI, kg/m^2^	23.9 ± 3.2 (12.2–34.9)	23.8 ± 3.3 (12.2–34.9)	24.0 ± 3.2 (17.6–29.6)	0.794
Ever smoker, %	129 (71.6%)	126 (88.1%)	3 (8.1%)	< 0.001
Smoking history, pack-years	31.0 ± 23.3 (0–150)	31.5 ± 23.3 (0–150)	8.3 ± 7.6 (0–15)	0.088
Diabetes, n	59 (32.8%)	47 (32.9%)	12 (32.4%)	1.000
Use of Statins or Sulfonylureas or Glinides, n	66 (36.7%)	52 (37.1%)	13 (35.1%)	1.000
FVC, L	2.6 ± 0.8 (0.9–4.8)	2.8 ± 0.8 (0.9–4.8)	1.8 ± 0.5 (0.9–2.8)	< 0.001
FVC, % predicted	76.3 ± 17.1 (31–110)	77.1 ± 17.1 (31–110)	72.8 ± 16.6 (34–106)	0.185
FEV_1_, L	2.1 ± 0.6 (0.8–3.9)	2.2 ± 0.6 (0.8–3.9)	1.3 ± 0.4 (0.8–2.3)	< 0.001
FEV_1_, % predicted	89.0 ± 18.6 (34–128)	89.3 ± 18.9 (34–128)	88.2 ± 17.7 (45–119)	0.753
FEV_1_/FVC, %	81.44 ± 8.97 (31–100)	80.53 ± 9.07 (31–100)	85.15 ± 7.60 (70–98)	0.007
TLC, L	4.73 ± 1.42 (1.91–8.40)	5.03 ± 1.31 (2.55–8.40)	3.10 ± 0.70 (1.91–4.70)	< 0.001
DL_CO_, ml/min/mm Hg	12.2 ± 5.2 (2.9–46.5)	12.8 ± 5.3 (2.9–46.5)	9.9 ± 3.7 (4.2–17.3)	0.005
DL_CO_, % predicted	69.6 ± 23.1 (20–149)	71.6 ± 23.3 (20–149)	61.1 ± 20.6 (25–102)	0.025
T4 level muscle CSA, cm^2^	96.0 ± 25.6 (36.8–163.5)	102.4 ± 23.4 (36.8–163.5)	71.4 ± 18.1 (33.4–111.3)	< 0.001
^a^T4 level muscle index, cm^2^/m^2^	35.7 ± 8.5 (13.2–55.9)	37.2 ± 8.2 (13.2–55.9)	30.0 ± 7.5 (17.9–45.0)	< 0.001
T12 Erector spinae muscle CSA, cm^2^	28.2 ± 9.1 (9.8–55.9)	29.4 ± 8.2 (13.2–55.9)	23.5 ± 7.5 (9.8–38.8)	0.001
^b^T12 muscle index, cm^2^/m^2^	10.5 ± 3.3 (3.8–23.2)	10.7 ± 3.3 (3.8–23.2)	9.9 ± 3.1 (4.5–15.8)	0.184
Follow-up time, months	37.9 ± 22.5 (0.1–91)	37.0 ± 21.9 (0.1–90.8)	41.3 ± 25.0 (0.4–91)	0.301
GAP Index score	3.3 ± 1.4 (0–7)	3.5 ± 1.4 (1–7)	2.8 ± 1.5 (0–6)	0.006
1-year survival, number, (%)	157 (87.2%)	124 (86.7%)	33 (89.2%)	0.688
2-year survival, number, (%)	138 (76.7%)	107 (74.8%)	31 (83.8%)	0.284

Continuous variables are presented as mean ± standard deviation (range) and categorical variables are presented as numbers (percentage)

*Abbreviations*: *BMI* Body Mass Index, *FEV1* Forced Expiratory Volume, *FVC* Forced Vital Capacity, *TLC* Total Lung Capacity, *DL*_*CO*_ diffusing capacity of carbon monoxide, *CSA* Cross Sectional Area, *GAP* gender, age, and physiologic variables

^a^T4 level muscle index = CSA of pectoralis, intercostalis, paraspinals, serratus, and latissimus muscles at T4 level/height^2^

^b^T12 level muscle index = CSA of erector spinae muscle at T12 level/height^2^

Compared with female patients, male patients had higher body mass indexes (BMI), a greater tendency to be smokers, higher FVC, L, forced expiratory volume (FEV_1_), L, and FEV_1_/FVC, DL_CO,_ higher GAP index score, higher T4_CSA_ and ESM_CSA_, higher T4MI (37.2 cm^2^/m^2^ vs. 30.0 cm^2^/m^2^, respectively, *P* <  0.001). FVC and FEV_1_ percentage of predicted value, and T12MI did not differ significantly between male and female patients.

Of 143 male patients with IPF, 53 (37.1%) died during the follow-up period. Respective 1-year survival and 2-year survival rates were 86.7 and 74.8%. Of 37 female patients with IPF, 15 (40.5%) died during the follow-up period. Respective 1-year survival and 2-year survival rates were 89.2 and 83.8%. One-year (*P* = 0.688) and two-year survival (*P* = 0.284) rates did not differ between male and female patients.

### Clinical characteristics and survival

We divided the patients into two groups according to CSA divided by height square to visualize the effects of lowest quartile CSA divided by height square. Male and female patients were divided and analyzed separately as male and female exhibited significant difference in muscle mass. And there is no defined cutoff point for defining sarcopenia in chest CT in T4 and T12 levels, we divided the patients into four groups according to T4MI and T12MI in male and female, respectively. And lowest quartile of T4MI or T12MI groups was defined as sarcopenia group.

When patients were stratified by T4_CSA_ divided by height square (T4MI) (*n* = 143); comparisons of clinical characteristics and survival between the lowest quartile of T4MI (sarcopenia) (Q4) and the rest (normal) group (Q1 + 2 + 3) are shown in Table 2. The cutoff value corresponds to the lowest quartile of T4MI (men: T4MI = 32.53 cm^2^/m^2^; women: T4MI = 24.25 cm^2^/m^2^, respectively). In male patients, Forced Vital Capacity (FVC)% predicted was lower and GAP index score was higher in the Sarcopenia group than in the normal group. Clinical characteristics, such as BMI, age, and smoking history did not differ between sarcopenia and normal groups. Respective 1-year survival rates of the Sarcopenia group and the normal group were 77.8 and 89.7% (*P* = 0.068). Respective 2-year survival rates of the Sarcopenia group and the normal group were 58.3 and 80.4% (*P* = 0.008). In female patients, although not statistically significant, the sarcopenia group showed a lower 2-year survival rate than that of the normal group (89.3% vs 66.7%, *P* = 0.109).

**Table 2:** Comparisons between Sarcopenia (lowest quartile of T4MI) (Q4) and the normal group (Q1 + Q2 + Q3) among men and women, respectively

Variable	Men(*n* = 143)			Women(*n* = 37)		
	Normal (*n* = 107)	Sarcopenia (*n* = 36)	*P*-value	Normal (n = 28)	Sarcopenia (*n* = 9)	*P*-value
Age, years	68.1 ± 7.9	70.8 ± 8.9	0.087	70.4 ± 8.2	71.6 ± 13.2	0.808
BMI, kg/m^2^	24.0 ± 3.1	23.3 ± 3.7	0.283	24.4 ± 3.1	22.7 ± 3.5	0.231
Ever smoker, %	92 (88.8%)	31 (86.1%)	0.336	3 (10.7%)	0 (0.0%)	0.592
Smoking history, pack-years	32.1 ± 23.9	29.7 ± 21.5	0.594	8.3 ± 7.6	0	n/a
Diabetes, n	36 (33.6%)	11 (30.6%)	0.839	8 (28.6%)	4 (44.4%)	0.432
Use of Statins or Sulfonylureas or Glinides, n	42 (39.3%)	11 (30.6%)	0.427	7 (25%)	6 (66.7%)	0.042
FVC, L	2.9 ± 0.7	2.62 ± 0.84	0.096	1.8 ± 0.5	1.7 ± 0.6	0.888
FVC, % predicted	78.9 ± 16.2	72.0 ± 18.9	0.040	73.8 ± 16.3	68.7 ± 18.1	0.516
FEV_1_, L	2.3 ± 0.6	2.14 ± 0.63	0.241	1.5 ± 0.4	1.5 ± 0.5	0.982
FEV_1_, % predicted	90.4 ± 18.0	86.1 ± 21.2	0.249	88.9 ± 17.8	85.3 ± 18.4	0.649
FEV_1_/FVC, %	79.8 ± 9.3	82.6 ± 8.1	0.117	84.6 ± 7.8	87.1 ± 6.6	0.409
DL_CO_, % predicted	73.7 ± 22.8	64.9 ± 23.9	0.065	59.8 ± 20.3	66.2 ± 22.6	0.551
T4 level muscle CSA, cm^2^	111.7 ± 17.4	74.7 ± 15.6	< 0.001	78.2 ± 14.8	50.3 ± 8.3	< 0.001
^a^T4 level muscle index, cm^2^/m^2^	40.6 ± 5.8	26.86 ± 5.01	< 0.001	33.0 ± 6.0	20.8 ± 2.0	< 0.001
T12 Erector spinae muscle CSA, cm^2^	31.8 ± 8.4	21.99 ± 7.46	< 0.001	26.0 ± 5.6	15.2 ± 5.8	< 0.001
^b^T12 muscle index, cm^2^/m^2^	11.6 ± 3.0	7.95 ± 2.85	< 0.001	11.0 ± 2.4	6.2 ± 2.2	< 0.001
Follow-up time, months	38.8 ± 21.0	31.6 ± 23.8	0.087	39.7 ± 21.6	46.2 ± 34.5	0.603
GAP Index score	3.3 ± 1.3	4.0 ± 1.6	0.012	2.7 ± 1.3	3.1 ± 2.1	0.610
1-year survival, number, (%)	96 (89.7%)	28 (77.8%)	0.068	26 (92.9%)	7 (77.8%)	0.205
2-year survival, number, (%)	86 (80.4%)	21 (58.3%)	0.008	25 (89.3%)	6 (66.7%)	0.109

Data are presented as number of patients (%) or mean ± standard deviation

*Abbreviations*: *Q* quartile, *BMI* Body Mass Index, *FEV1* Forced Expiratory Volume, *FVC* Forced Vital Capacity, *TLC* Total Lung Capacity, *DL*_*CO*_ diffusing capacity of carbon monoxide, *CSA* Cross Sectional Area, *GAP* gender, age, and physiologic variables

Cutoff values for lower quartile in men and women are 32.53 cm^2^/m^2^ and 24.25 cm^2^/m^2^, respectively

^a^T4 level muscle index = CSA of pectoralis, intercostalis, paraspinals, serratus, and latissimus muscles at T4 level/height^2^

^b^T12 level muscle index = CSA of erector spinae muscle at T12 level/height^2^

**Fig. 3:**
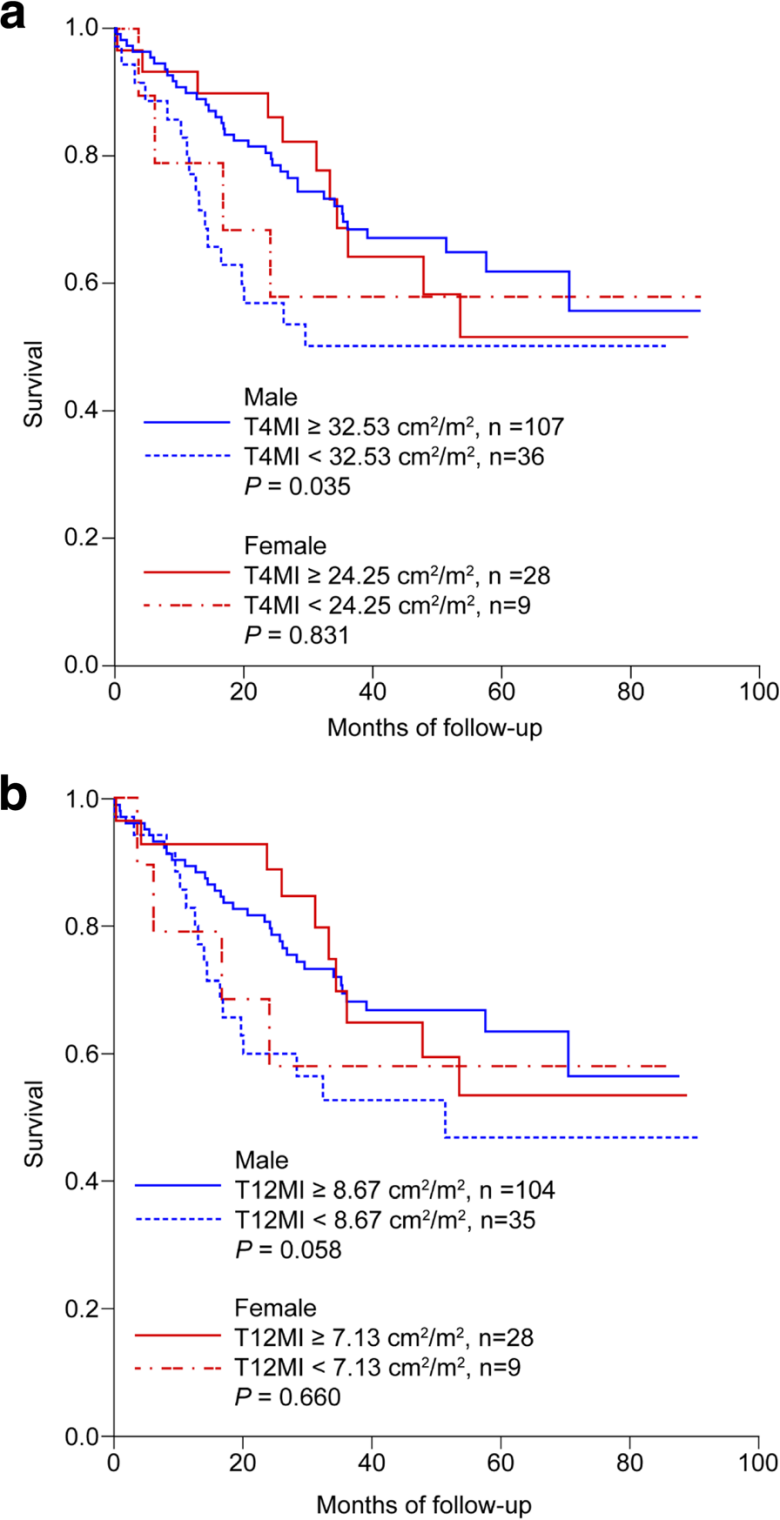
**a** Kaplan–Meier survival curves stratified by the cross-sectional area of pectoralis, intercostalis, paraspinals, serratus, and latissimus muscles at the T4 level in male and female patients . The cutoff value corresponds to the lowest quantile of the cross-sectional area of muscle at the T4 level/height^2^ (T4 muscle index, T4MI) (Male: T4MI = 32.53 cm^2^/m^2^, *n* = 108/35 respectively / Female: T4MI = 24.25 cm^2^/m^2^, *n* = 28 / 9 respectively). **b** Kaplan–Meier survival curves stratified by the cross-sectional area of the erector spinae muscles at the T12 level in male and female patients. The cutoff value corresponds to the lowest quantile of the cross-sectional area of erector spinae muscle/height^2^ (T12 muscle index, T12MI). (Male: T12MI = 8.67 cm^2^/m^2^, *n* = 104/35 respectively / Female: T12MI = 7.13 cm2/m2, *n* = 28 / 9 respectively)

Kaplan-Meier survival curves stratified by the sarcopenia and the normal group in male and female patients are shown in Fig. 3a. Male patients in the sarcopenia group exhibited a significantly lower survival rate (*P* = 0.035) than those of the normal group. There was no significant difference in survival between the sarcopenia and the normal group (*P* = 0.831) in Kaplan-Meier survival curves among female patients.

Furthermore, patients were stratified by the ESM_CSA_ divided by height square (T12MI) (*n* = 139); comparisons of clinical characteristics and survival between the lowest quartile of T12MI (Sarcopenia) (Q4) and the rest (normal) group (Q1 + 2 + 3) are shown in Table 2. The cutoff value corresponds to the lowest quartile of T12MI (men: T12MI = 8.67 cm^2^/m^2^; women: 7.13 cm^2^/m^2^, respectively). In male patients, patients with sarcopenia exhibited lower survival although the difference was not statistically significant in men (*P* = 0.058). Respective 1-year survival rates of the sarcopenia group and normal groups were 82.8 and 89.4% (*P* = 0.305). Respective 2-year survival rates of the sarcopenia and normal groups were 60.0 and 80.8% (*P* = 0.013). In female patients, although not statistically significant, the sarcopenia group showed lower a 2-year survival rate than that of the normal group (92.3% vs 66.7%, *P* = 0.058).

Kaplan-Meier survival curves stratified by sarcopenia group and normal group in male and female patients are shown in Fig. 3b. Male patients in the sarcopenia group exhibited a lower survival rate (*P* = 0.058) than that of the normal group, although this was not statistically significant. There was no significant difference in survival rates between the normal and the sarcopenia groups (*P* = 0.660) in Kaplan-Meier survival curves among female patients.

### Cox proportional hazards analyses

The relationships between all-cause mortality and clinical parameters, including T4MI and T12MI, were evaluated with Cox proportional hazards analysis (Table 3). Univariate analysis showed that lower BMI, lower FVC% predicted value, lower FEV_1_% predicted value, lower DL_CO_% predicted value, lower T4MI (HR, 0.965; 95% CI, 0.937–0.993; *P* = 0.015), lower T12MI (HR, 0.910; 95% CI, 0.841–0.984; *P* = 0.019), and a higher GAP index score were significantly correlated with all-cause mortality.

**Table 3:** Clinical factors associated with all-cause mortality in men and women patients (univariate analysis)

Variable	Total (*n* = 180)		Men (n = 143)		Women (n = 37)	
	HR (95% CI)	*P* value	HR (95% CI)	P value	HR (95% CI)	P value
Age, yr	1.033 (1.001–1.064)	0.037	1.012 (0.978–1.047)	0.507	1.098 (1.036–1.164)	0.002
Sex, Female	1.031 (0.581–1.831)	0.916				
BMI, kg/m^2^	0.900 (0.833–0.971)	0.007	0.861 (0.789–0.938)	0.001	1.044 (0.895–1.219)	0.583
Smoking history, pack-years	1.004 (0.993–1.015)	0.488	1.005 (0.994–1.016)	0.406	0.757 (0.259–2.211)	0.610
Diabetes, n	1.495 (0.922–2.425)	0.103	1.275 (0.731–2.222)	0.392	2.521 (0.907–7.012)	0.076
Use of Statins or Sulfonylureas or Glinides, n	0.962 (0.588–1.576)	0.879	0.944 (0.538–1.655)	0.840	0.964 (0.341–2.725)	0.945)
FVC % predicted	0.958 (0.944–0.973)	< 0.001	0.961 (0.945–0.977)	< 0.001	0.943 (0.907–0.982)	0.004
FEV1, % predicted	0.973 (0.960–0.986)	< 0.001	0.975 (0.961–0.989)	0.001	0.964 (0.932–0.996)	0.030
DLCO % predicted	0.972 (0.959–0.985)	< 0.001	0.969 (0.955–0.984)	< 0.001	0.975 (0.938–1.012)	0.183
TLC, % predicted	0.970 (0.950–0.990)	0.004	0.970 (0.950–0.990)	0.004	0.944 (0.882–1.009)	0.091
^a^T4 level muscle index, cm^2^/m^2^	0.965 (0.937–0.993)	0.015	0.958 (0.926–0.991)	0.013	0.972 (0.904–1.044)	0.433
^b^T12 level muscle index, cm^2^/m^2^	0.910 (0.841–0.984)	0.019	0.908 (0.831–0.992)	0.033	0.917 (0.772–1.089)	0.322
GAP Score	1.356 (1.137–1.619)	0.001	1.366 (1.112–1.678)	0.003	1.403 (0.977–2.014)	0.067

Data are presented as Hazard Ratios (95% Confidence Intervals)

*Abbreviations*: *BMI* Body Mass Index, *FEV1* Forced Expiratory Volume, *FVC* Forced Vital Capacity, *TLC* Total Lung Capacity, *DL*_*CO*_ diffusing capacity of carbon monoxide, *CSA* Cross Sectional Area, *GAP* gender, age, and physiologic variables

^a^T4 level muscle index = CSA of pectoralis, intercostalis, paraspinals, serratus, and latissimus muscles at T4 level/height^2^

^b^T12 level muscle index = CSA of erector spinae muscle at T12 level/height^2^

Multivariate Cox proportional hazards analyses were performed to compare the contributions of these indices (Table 4). Stepwise Cox proportional hazards analysis demonstrated that lower BMI (HR, 0.885; 95% CI, 0.806–0.972; *P* = 0.010) and lower T4MI (HR, 0.955; 95% CI, 0.913–0.998; *P* = 0.041) were risk factors for all-cause mortality. Lower T12MI (HR, 0.980; 95% CI, 0.856–1.121; *P* = 0.766) was not a significant risk factor for all-cause mortality after multivariate analysis. Higher GAP index score was a significant risk factor for all-cause mortality (HR, 1.450; 95% CI, 1.169–1.760; *P* = 0.001).

**Table 4:** Clinical factors associated with all-cause mortality in the entire cohort (multivariate analysis)

Variables	HR	95% CI	P value
BMI, kg/m^2^	0.885	0.806–0.972	0.010
Smoking history, pack-years	0.993	0.977–1.009	0.367
^a^T4 level muscle index, cm^2^/m^2^	0.955	0.913–0.998	0.041
^b^T12 level muscle index, cm^2^/m^2^	0.980	0.856–1.121	0.766
GAP Score	1.450	1.169–1.760	0.001

Data are presented as Hazard Ratios (95% Confidence Intervals)

*Abbreviations*: *BMI* Body Mass Index, *GAP* gender, age, and physiologic variables

^a^T4 level muscle index = CSA of pectoralis, intercostalis, paraspinals, serratus, and latissimus muscles at T4 level/height^2^

^b^T12 level muscle index = CSA of erector spinae muscle at T12 level/height^2^

## Discussion

To our knowledge, this study is the first to demonstrate that quantitatively analyzing muscles with chest CT can be useful in predicting the prognosis in patients with IPF. Our study showed that low T4MI is a risk factor for all-cause mortality in IPF patients, along with established and reported prognostic predictors.

There can be several reasons for the relationship between low T4MI with poor survival. First, muscles at the T4 level are involved with breathing; quantitative differences could result in altered performance, thus resulting in altered outcomes. Second, decreased lung function and symptoms of dyspnea could be reasons for reduced muscle mass. Reduced physical activity due to decreased lung function and dyspnea, is well-documented and this might result in reduced muscle mass [19, 20]. However, it is unclear whether lower muscle mass led to increased disease severity, or whether increased disease severity led to lower muscle mass. Further studies investigating this mechanism are thus required.

Measurement of T4_CSA_ has several advantages, compared with parameters described in other reports pertaining to the skeletal muscles, [12] such as the psoas [14] and mid-thigh muscles [21]. Psoas muscle CSA may be affected by positions of upper extremities during scanning. Notably, other skeletal muscles require additional scans and X-ray exposure for analysis; however, a patient may not be able to undergo another evaluation of sarcopenia because of poor health. Without any additional radiation exposure, CSA analysis through existing chest CT scans provides an additional and important objective index of disease severity and future prognosis in patients with IPF.

In our study, Kaplan–Meier survival analysis for male patients with IPF who exhibited lower T12MI values demonstrated a significantly lower 2-year survival rate; however, it was not significant in multivariate Cox proportional hazards analysis. In the CT-based evaluation of sarcopenia, transverse skeletal muscle index at L3, which includes ESM, correlates well with whole-body composition measurements performed with dual-energy X-ray absorptiometry [22]. Similarly, there is possibility that measuring ESM at the level of T12 could have clinical significance if CSA of muscles involved in breathing are included in the analysis of CSA at the level of T12, considering the proximity of L3. More studies are needed to clarify the relationship between muscle CSA at the level of T12 and outcomes in IPF patients.

Many individual clinical variables have been shown to predict survival in IPF [23–25]. The effect of muscle mass on IPF prognosis was unknown. However, several studies [6, 14, 15] suggested that thoracic muscle CSA is related to physical activity, quadriceps volume, and health-related quality of life in other lung diseases, such as lung transplantation, chronic obstructive lung disease, and non-small-cell lung cancer. Similarly, Izawa et al. suggested a relationship between respiratory muscle strength and sarcopenia in elderly cardiac patients [26]. Muscle mass involved in respiratory diseases may be also important for IPF patients; indeed, our results showed that skeletal muscle mass evaluated using chest CT can be a prognostic factor in IPF.

Our study has certain limitations. First, the study was retrospective study and the sample size was small, from a single center, and involved no replication cohort. Only Korean population were included in this study. This cut-off values cannot be used in other races or population considering that the cutoff for sarcopenia can differ among different races [27]. However, the validity of reported prognostic factors, such as the GAP score, was confirmed in this study population, which supports the present findings. In fact, the variables which were not statistically significant in the analysis of female patients could have been influenced by the small sample size. Second, a standard method for measuring CSA of the thoracic muscles has not yet been established. As reports regarding measurement of thoracic muscles are currently undergoing publication, we believe that a standard method for measuring CSA of thoracic muscles will soon be established. Third, the physical activity levels of all subjects were not directly evaluated. It is possible that there may be relationship to physical inactivity and lower muscle mass. We could not include data regarding physical function testing as these were not available at the time of analysis. However, based on other reports, [4, 5, 13] we suspect that skeletal muscles of the chest may reflect both physical activity and physiological parameters. Further analysis is needed to verify these assumptions.

## Conclusions

In conclusion, low T4_CSA_, normalized for stature, obtained from a single-slice axial chest CT image may be a strong risk factor for all-cause mortality in patients with IPF. Without additional radiation exposure, CSA analysis via existing chest CT scans provides an important index reflecting future prognosis in patients with IPF. This might aid in selection of an optimal treatment and enable early intervention to maintain muscle mass and improve prognosis. Close observation is needed in IPF patients with a lower skeletal muscle mass and early referral to lung transplantation program can be suggested in these patients if they fulfill the criteria for lung transplantation. Furthermore, referring to pulmonary rehabilitation can be beneficial in these patients. Additional studies are required to determine optimum reference values for predicting IPF-specific outcomes.

## Additional file

Additional file 1: **Table S1.** Comparisons between sarcopenia group (lower quartile of T12MI) (Q4) and the normal group (Q1 + Q2 + Q3) among men and women, respectively. (SAV 93 kb)

## Abbreviations

BMI: Body mass index
COPD: Chronic obstructive pulmonary disease
CSA: Cross-sectional area
CT: Computed tomography
DLCO: Diffusing capacity of carbon monoxide
ESM: Erector spinae muscle
ESMCSA: Cross-sectional area measured at erector spinae muscle
FEV1: Forced expiratory volume
FVC: Forced vital capacity
GAP index: Gender-Age-Physiology Index
IPF: Idiopathic pulmonary fibrosis
MI: Muscle index
SD: Standard deviation
T4CSA: Cross-sectional area measured at T4
TLC: Total lung capacity
